# Fungal Pulmonary Valve Endocarditis Masquerading as a Pulmonary Embolism

**DOI:** 10.1155/2015/850852

**Published:** 2015-02-22

**Authors:** Kevin B. Ricci, Peter H. U. Lee, Michael Essandoh, Ahmet Kilic

**Affiliations:** ^1^Department of Surgery, The Ohio State University Wexner Medical Center, Columbus, OH 43201, USA; ^2^Department of Cardiac Anesthesia, The Ohio State University Wexner Medical Center, Columbus, OH 43201, USA

## Abstract

Septic pulmonary emboli (SPE) can be a difficult clinical entity to distinguish from thromboembolic pulmonary embolism (TPE) in a patient with history of IV drug abuse (IVDA). We present a case of a patient who presented with failure to thrive and presumed diagnosis of recurrent PE that ultimately was discovered to have fungal pulmonary valve endocarditis resulting in a right ventricular outflow obstruction. This required replacement of the pulmonary valve and repair of the right ventricular outflow tract. This case highlights difficulty in differentiating pulmonary valve endocarditis with septic emboli from chronic PE in a patient with a complex medical history.

## 1. Case Description

**Figure 1:**
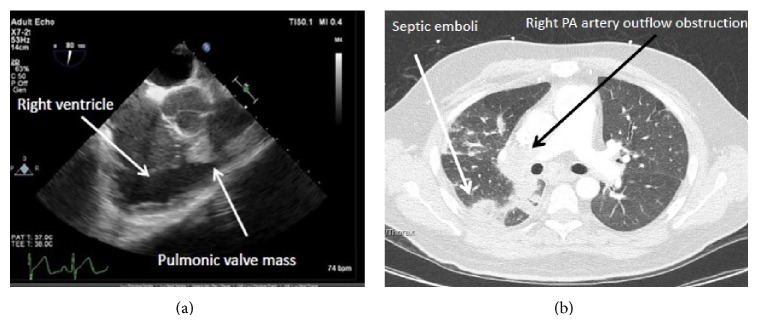
(a) Transesophageal echocardiogram demonstrating a large mass in the right ventricular outflow tract during diastole. (b) CT angiography of the chest showing a right PA artery outflow obstruction and septic pulmonary emboli.

A 38-year-old male presented to our institution from an outside hospital with a four-month history of failure to thrive with a sixty-pound weight loss, progressive shortness of breath (SOB), and productive cough. Initial evaluation at the outside hospital was concerning for chronic pulmonary embolism, which was treated with anticoagulation therapy (Figure 1). A repeat transthoracic echocardiogram at our institution demonstrated a large bilobed ventricular valve mass obstructing ventricular outflow tract as well as pulmonary insufficiency (Figure 1). Of note he endorsed a history of intravenous drug abuse and was known to be hepatitis B and C positive. Blood cultures were obtained and subsequently were positive for* Candida* species. Given this information the patient was diagnosed with septic emboli from fungal pulmonary valve endocarditis. The patient was medically optimized to undergo pulmonary valve replacement.

**Figure 2:**
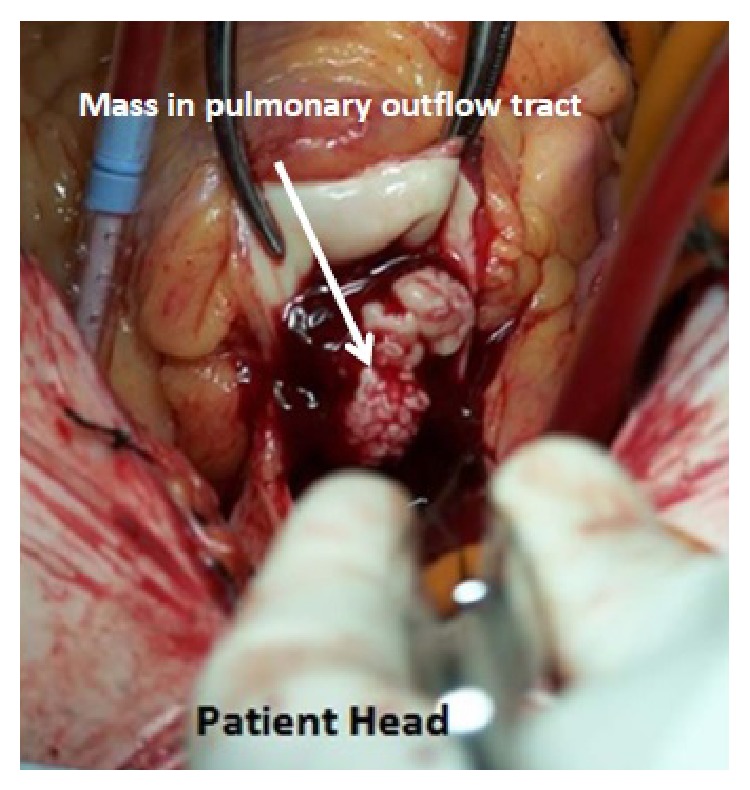
View from median sternotomy showing large bilobed mass in right ventricular outflow tract (divided open) with destruction of pulmonary valve before excision and repair.

He was taken to the operating suite where a median sternotomy was performed. After a pericardiotomy and the development of a pericardial well, it became apparent that there was an aneurysmal shape to the right ventricular outflow tract and thickening of the pulmonary artery. Cardiopulmonary bypass was initiated and the pulmonary artery was divided proximal to the bifurcation, revealing a large 4 × 3 cm and 3 × 2 cm bilobed mass involving all three leaflets (Figure 2). The infected tissue was excised and a 27 mm Trifecta valve was sutured in place (St. Jude Medical, St. Paul, Minnesota). The pulmonary outflow tract was reconstructed with bovine pericardium using a running 5-0 Prolene suture. His postoperative course was unremarkable and he was discharged on postoperative day 9 with 6-week course of oral fluconazole.

## 2. Discussion

Recurrent SPE is a rare but previously described complication from right sided infective endocarditis [1]. This particular case highlights the difficulty of distinguishing recurrent TPE from SPE. The initial diagnosis of TPE was consistent with patient's nonspecific symptoms of SOB and productive cough in the absence of positive bacterial blood cultures. However, it should be recognized that fungal species are a rare but known culprit of infective endocarditis in patients with immune compromised states or a history of IVDA, which are not easily identified on routine blood cultures. Furthermore, septic emboli may be the first and only symptom of fungal endocarditis, which can have severe complications [2]. As a result, a high index of clinical suspicion must remain present for infective endocarditis in a patient with a history of IVDA. In this case, failing to couple echocardiographic imaging alongside appropriate microbiology data led to the misdiagnosis of the patient's condition and a delay in definitive diagnoses and treatment.

Additionally, SPEs can be misidentified as TPEs on CT scanning. Both conditions can be identified by filling defects in the pulmonary vasculature on a CTPE study. A systematic review by Zhao and colleagues of 168 cases of SPE and corresponding CT imaging demonstrated unique characteristics of SPEs that included multiple bilateral nodules and patchy infiltrates that frequently occur near the pleura. Bilateral pleural effusions were also identified in approximately 30% of patients [3]. Although these findings do not exclude TPE based on CT imaging, combining the patient's history of IVDA, symptoms, and other imaging data will help accurately determine the diagnosis. This study also demonstrated that 52% of patients had evidence of right heart vegetations echocardiography, with the tricuspid valve most commonly involved. Positive findings on echocardiography with consistent CT imaging are highly supportive of the diagnoses of SPE [3]. This case demonstrates the importance of integrating all available data in the context of the patient's history with particular awareness of the subtle difference of SPEs from TPEs on CT imaging.
